# Pregabalin and duloxetine combination for painful diabetic neuropathy: a systematic review and meta-analysis

**DOI:** 10.3389/fendo.2026.1750441

**Published:** 2026-03-11

**Authors:** Yifan Shi, Yuyang Chen, Hengxia Zhao

**Affiliations:** 1 The Fourth Clinical Medical School of Guangzhou University of Chinese Medicine, Shenzhen, China; 2 Department of Endocrinology, Shenzhen Traditional Chinese Medicine Hospital, Shenzhen, Guangdong, China

**Keywords:** duloxetine, efficacy, meta-analysis, painful diabetic neuropathy, pregabalin, safety

## Abstract

**Background:**

Diabetic neuropathy is one of the most common complications of diabetes, affecting about half of all people with the disease. Among these, 30%-50% experience nerve-related pain, characterized by abnormal sensations, burning, or stabbing pain, a condition known as painful diabetic neuropathy (PDN). PDN not only severely impairs quality of life but is also closely associated with sleep disturbances, depression or anxiety, and foot complications. Together, these problems substantially increase healthcare costs and place a considerable economic burden on both families and society.

**Methods:**

We systematically searched multiple databases from their inception to 1 November 2025 to identify randomized controlled trials evaluating pregabalin combined with duloxetine for the treatment of painful diabetic neuropathy. The methodological quality and risk of bias of the included trials were assessed using the Cochrane risk-of-bias tool (version 2.0). Statistical analyses were performed with RevMan 5.4.

**Results:**

Three randomized trials involving a total of 471 patients were included. In two studies that could be pooled, combination therapy produced significantly greater pain relief than monotherapy (MD=-1.82, 95%CI=-2.10, -1.54, *P*<0.00001). For secondary continuous outcomes reported in single studies, all results favored the combination, pain intensity on the visual analogue scale (VAS, MD=-1.42, 95%CI=-1.83, -1.01, *P*<0.00001), brief pain inventory-modified short form (BPI-MSF, MD=-1.46, 95%CI=-2.35, -0.57, *P* = 0.001), and neuropathic pain symptoms on the pain detect questionnaire (PDQ, MD=-3.00, 95%CI=-5.55, -0.45, *P* = 0.02). The proportion of patients achieving at least 50% pain reduction was also higher with the combination than with duloxetine 120 mg alone (RR = 1.81, 95%CI=1.17, 2.81, *P* = 0.008). In contrast, there were no significant differences between combination therapy and monotherapy in the overall risk of adverse events (RR = 1.10, 95%CI=0.84, 1.46, *P* = 0.48) or in key individual adverse effects, including somnolence (RR = 0.79, 95%CI=0.30, 2.08, *P* = 0.63) and nausea/vomiting (RR = 2.02, 95%CI=0.77, 5.27, *P* = 0.15). The certainty of evidence ranged from very low to low for most outcomes (GRADE).

**Conclusion:**

Low-certainty evidence suggests that pregabalin plus duloxetine may improve short-term pain scores compared with monotherapy in painful diabetic neuropathy. Safety outcomes remain uncertain due to few trials and imprecision.

**Systematic review registration:**

https://www.crd.york.ac.uk/prospero/, identifier CRD420251179997.

## 1 Introduction

Diabetes is a long-term metabolic disease characterized by persistently elevated blood glucose levels. According to the 11th edition (2025) of the International Diabetes Federation (IDF) Diabetes Atlas (1), an estimated 589 million adults aged 20–79 years worldwide are living with diabetes, of whom about 252 million remain undiagnosed. By 2050, the number of adults with diabetes is projected to rise to 853 million. This rapid increase places a substantial economic burden on health systems and society. The IDF estimates that annual diabetes-related healthcare expenditure has reached at least 1 trillion US dollars, representing an increase of about 338% compared with 17 years ago (2). Against this background, diabetes-related complications, particularly those that cause chronic pain and disability, deserve close attention.

Diabetic neuropathy (DN) is the most common complication of diabetes, affecting approximately 50% of people with the disease (3, 4). Clinically, DN is characterized by chronic loss of sensation that usually begins in the feet and hands and gradually progresses toward the trunk, often in a distal-to-proximal pattern (5). Its development and progression are influenced by multiple factors, including duration of diabetes, quality of blood glucose control, age, obesity, abnormal blood lipids, insulin resistance, lifestyle behaviors, cardiovascular health, and genetic susceptibility (6–8). Among individuals with DN, 30%-50% experience nerve-related pain, described as abnormal sensations, burning, or stabbing pain, this condition is referred to as painful diabetic neuropathy (PDN) (9). Despite its clinical importance, the risk factors for PDN are still not fully known. Available evidence suggests that female sex, longer duration of diabetes, greater severity of neuropathy, impaired kidney function, higher glycated hemoglobin (HbA1c) levels, and increased body mass index (BMI) are potential contributors (10–14). Recent systematic reviews and clinical studies indicate that PDN is common both among patients with DN and in the overall diabetic population, and its prevalence rises with longer disease duration. PDN substantially reduces quality of life and is closely associated with sleep disturbances, depression or anxiety, and a higher risk of foot complications such as ulcers, infections, and non-traumatic lower-limb amputations. These problems markedly increase healthcare costs and impose a considerable economic burden on patients, their families, and society (15–17).

Current reviews and clinical guidelines agree that the main first-line medicines for PDN are duloxetine, pregabalin, gabapentin, and amitriptyline, which show broadly similar effectiveness. Treatment choice is usually individualized according to comorbid conditions and potential side effects, and drugs may be used sequentially or in combination. Routine use of opioid analgesics is not recommended. In addition, topical capsaicin 8% patches and lidocaine patches are supported by evidence in selected patient groups (18, 19). For treatment-resistant PDN, evidence for non-invasive neuromodulation techniques and spinal cord stimulation has grown markedly in recent years, however, short follow-up durations and relatively small sample sizes still limit the strength of recommendations for these interventions (20, 21).

Among these medicines, pregabalin and duloxetine are the only drugs approved by the US Food and Drug Administration (FDA) for painful diabetic neuropathy. They act through different mechanisms, pregabalin mainly targets specific receptors on nerve cells (α2-δ subunits of voltage-gated calcium channels), duloxetine increases the availability of certain neurotransmitters in the central nervous system (22, 23). Duloxetine has strong evidence supporting its pain-relieving effect in PDN. A daily dose of 60 mg is usually sufficient to balance efficacy and tolerability, and doses above 60 mg per day generally do not provide additional pain relief but may increase side effects (24). Randomized trials and systematic reviews have shown that duloxetine can reduce pain intensity and improve sleep and quality of life. The most common side effects include nausea, somnolence, and dry mouth. Blood pressure should be monitored, and potential drug interactions need attention, particularly with monoamine oxidase inhibitors and strong CYP1A2 or CYP2D6 inhibitors (25, 26). Pregabalin also has well-established benefits for PDN. The recommended starting dose is 150 mg per day in divided doses, which can be increased to 300 mg per day within about one week according to pain control and tolerability (27). Dose adjustment is required in patients with reduced kidney function. Typical side effects include dizziness, somnolence, fluid retention, and weight gain. Reviews have shown that pregabalin provides consistent pain relief across various types of nerve-related pain, supporting its role as a key option in the management of PDN (28).

However, standard doses of pregabalin or duloxetine alone provide meaningful pain relief in only about 40% of patients with PDN (29). For patients who show only a partial response to either drug at usual doses, combining standard doses of duloxetine and pregabalin may offer better pain control and tolerability than increasing either medicine to its maximum dose, although this approach may be limited by side effects (30). Because pregabalin and duloxetine act through different but potentially complementary mechanisms (31, 32), their combination may produce an additive clinical effect on nerve-related pain compared with either drug alone, thereby enhancing pain relief. Therefore, our study aimed to evaluate the effectiveness and safety of combining pregabalin with duloxetine for the treatment of painful diabetic neuropathy. By comparing clinical outcomes between combination therapy and monotherapy, we sought to determine whether the combined regimen provides superior pain relief and higher response rates. In addition, we examined the safety profile of the combination to clarify whether enhanced efficacy is achieved without a substantial increase in adverse effects. These findings are intended to offer clinically relevant evidence to support decision-making in the management of painful diabetic neuropathy.

## 2 Methods

### 2.1 Study design

This systematic review was conducted in accordance with the Preferred Reporting Items for Systematic Reviews and Meta-Analyses (PRISMA) guideline (33). The protocol was prospectively registered in PROSPERO, registration number CRD420251179997, and the completed PRISMA checklist is provided in **Supplementary Table 1**.

### 2.2 Literature search strategy

We performed a comprehensive literature search in multiple databases, including PubMed, Embase, Web of Science, the Cochrane Library, China National Knowledge Infrastructure (CNKI), Wanfang, and VIP. All databases were searched from their inception to 1 November 2025, without language restrictions. Both medical subject headings and free-text terms were used, and the detailed search strategies for each database are provided in **Supplementary Table 2**.

### 2.3 Inclusion and exclusion criteria

Inclusion criteria: (1) randomized controlled trials; (2) participants with a clear diagnosis of painful diabetic neuropathy; (3) clinical studies evaluating the effectiveness or safety of pregabalin combined with duloxetine for PDN; (4) original studies that used standardized measures of treatment benefit and adverse events, such as pain relief and the occurrence of side effects. Exclusion criteria: (1) not randomized controlled trials; (2) the intervention is not pregabalin combined with duloxetine, or the comparison is not pregabalin or duloxetine alone; (3) the study population did not consist solely of patients with PDN; (4) the data were incomplete or could not be used for analysis.

### 2.4 Data extraction

Two reviewers independently screened the literature and extracted data using a standardized form, with cross-checking to ensure accuracy. Any disagreements were resolved through discussion or, if necessary, consultation with a third reviewer. Extracted information included first author, year of publication, sample size, mean age of participants, details of the intervention and comparison, treatment duration, and outcome measures. The main outcomes were overall treatment response, numerical rating scale (NRS) scores, visual analogue scale (VAS) scores, Brief Pain Inventory-Modified Short Form (BPI-MSF) scores, pain detect questionnaire (PDQ) scores, and the incidence of adverse events.

### 2.5 Risk of bias assessment

We assessed the methodological quality and risk of bias of the included randomized controlled trials using the Cochrane Risk of Bias 2.0 (RoB 2.0) tool (34). This tool evaluates five domains. First, bias arising from the randomization process was judged by examining how the random sequence was generated, for example, use of a computer-generated random number table, whether allocation was adequately concealed, and whether baseline characteristics were balanced between groups. Second, bias due to deviations from intended interventions was assessed by considering whether blinding was implemented, whether lack of blinding might have led to differences in co-interventions or treatment adherence, and whether an intention-to-treat approach was used in the analysis, to determine whether the actual interventions received differed meaningfully from the original study design.

Third, bias due to missing outcome data was evaluated by comparing the rates and reasons for loss to follow-up or withdrawal between groups, assessing whether missingness was related to the outcomes, and judging whether appropriate methods were used to handle missing data, in order to decide whether this could have introduced systematic error. Fourth, bias in the measurement of outcomes was assessed according to the type of outcome, including whether the measurement tools were reliable and standardized, whether outcome assessors were blinded, whether measurement methods and timing were consistent across groups, and whether knowledge of group assignment might have influenced outcome assessment. Fifth, bias in the selection of the reported result was examined by comparing published reports with study protocols or trial registrations, to identify any selective reporting of outcomes or analyses. Each domain, as well as the overall study, was rated as having low risk of bias, some concerns, or high risk of bias.

### 2.6 Certainty of evidence

We evaluated the certainty of the evidence using the Grading of Recommendations, Assessment, Development and Evaluation (GRADE) approach (35). As recommended, evidence from randomized controlled trials was initially rated as high certainty and could then be downgraded based on five domains: risk of bias, inconsistency, indirectness, imprecision, and publication bias. After considering these factors, the overall certainty for each outcome was classified as high, moderate, low, or very low. All assessments were performed independently by two reviewers, with disagreements resolved through discussion and, when necessary, adjudication by a third reviewer.

### 2.7 Statistical analysis

Statistical analyses were performed using RevMan 5.4. For continuous outcomes, mean differences (MDs) were calculated, and for dichotomous outcomes, risk ratios (RRs) were used. Heterogeneity across studies was assessed with the *I²* statistic and the Q test. An *I²* value greater than 50% or a Q test *P* < 0.10 was considered to indicate substantial heterogeneity, in which case a random-effects model was applied, otherwise, a fixed-effect model was used.

## 3 Results

### 3.1 Literature search and screening results

**Figure 1:**
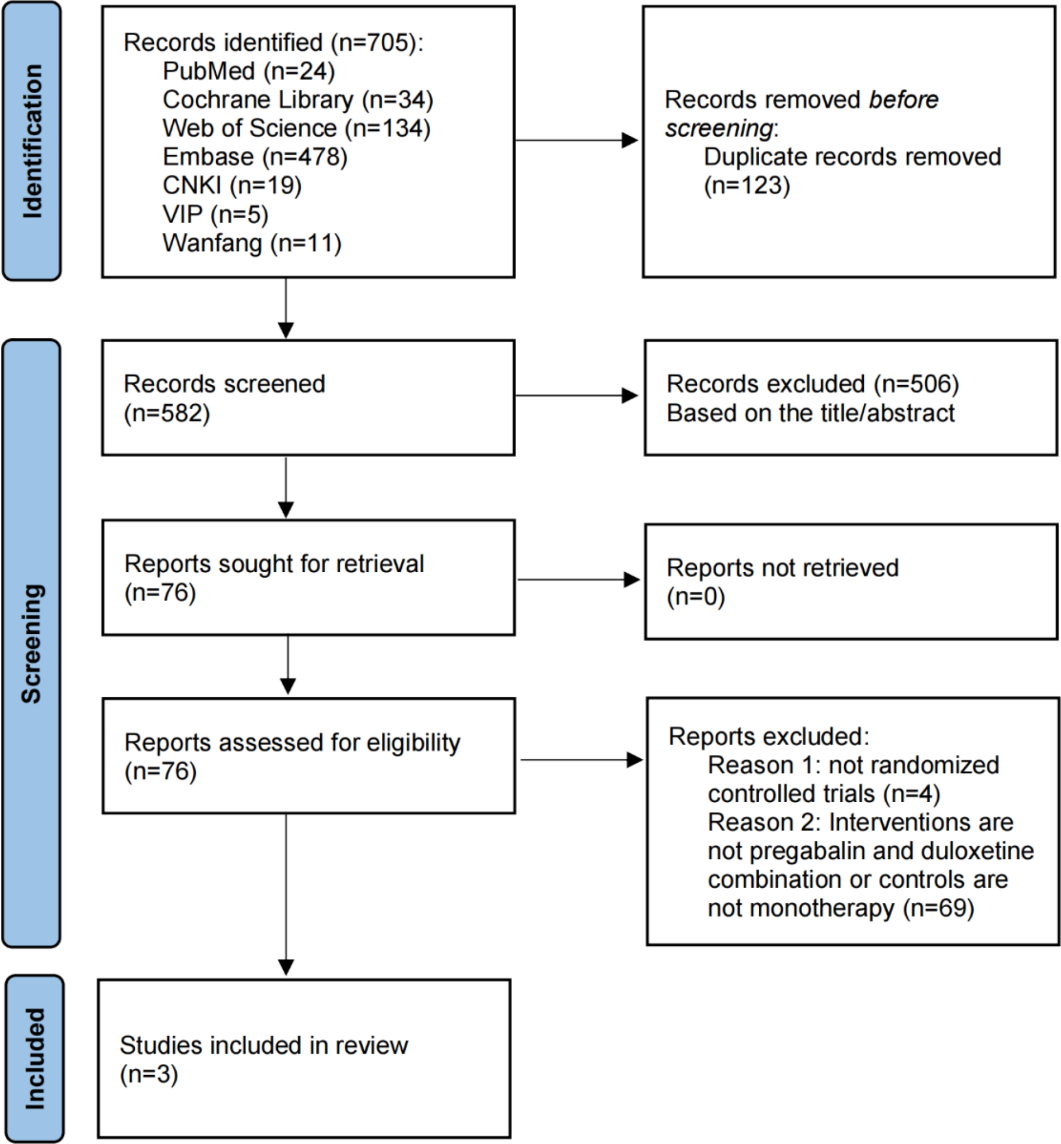
Flowchart of study selection.

A total of 705 records were identified through searches of PubMed, Embase, Web of Science, the Cochrane Library, CNKI, Wanfang, and VIP. After removing 123 duplicates, 582 records remained. These were screened by title, abstract, and then full text against the predefined inclusion and exclusion criteria. In the end, three studies met the criteria and were included in the review (36–38). The study selection process is summarized in **Figure 1**.

### 3.2 Characteristics of included studies and quality assessment

The main characteristics of the included randomized controlled trials are summarized in **Table 1**. In total, 471 patients with a confirmed diagnosis of painful diabetic neuropathy were enrolled. In all three studies, the intervention consisted of combination therapy with pregabalin and duloxetine, while the control groups received either pregabalin or duloxetine alone (36–38).

**Table 1:** Baseline characteristics of the included studies.

Included studies	Sample size		Mean age, years		Intervention measure		Treatment periods	Outcome measures
	Intervention group	Comparison group	Intervention group	Comparison group	Intervention group	Comparison group		
QI 2022	51	51	66.38 ± 6.23	67.96 ± 6.22	Pregabalin (75mg, bid), plus Duloxetine (30mg, bid)	Duloxetine (30mg, bid)	4 weeks	Efficacy, NRS, VAS, adverse events
Tesfaye 2013	169	170	61.0 ± 9.78	61.2 ± 10.46	Pregabalin (300mg), plus Duloxetine (60mg)	Pregabalin (600mg), or Duloxetine (120mg)	16 weeks	Pain relief ≥50%, ≥30%, ≥2 points, other BPI-MSF items, the Clinical Global Impression of Improvement scores, the Patient Global Impression of Improvement scores, NPSI and its 5 subscores, HADS, adverse events
Saxena 2024	15	15	55.67 ± 9.98	53.53 ± 11.58	Pregabalin (75mg, bid), plus Duloxetine (30mg, bid)	Pregabalin (75mg, bid)	4 weeks	NRS, PDQ, BPI-MSF, SF12, adverse events

**Figure 2:**
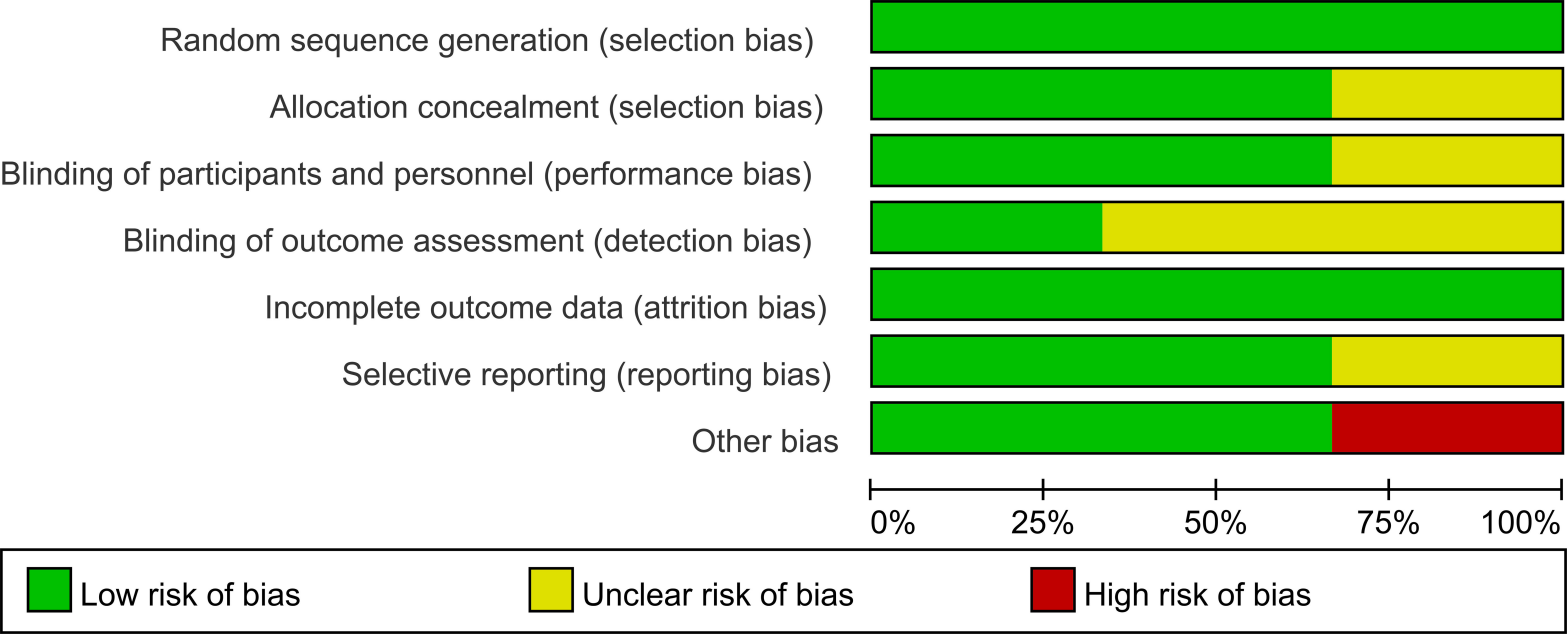
Quality evaluation of the included studies.

**Figure 3:**
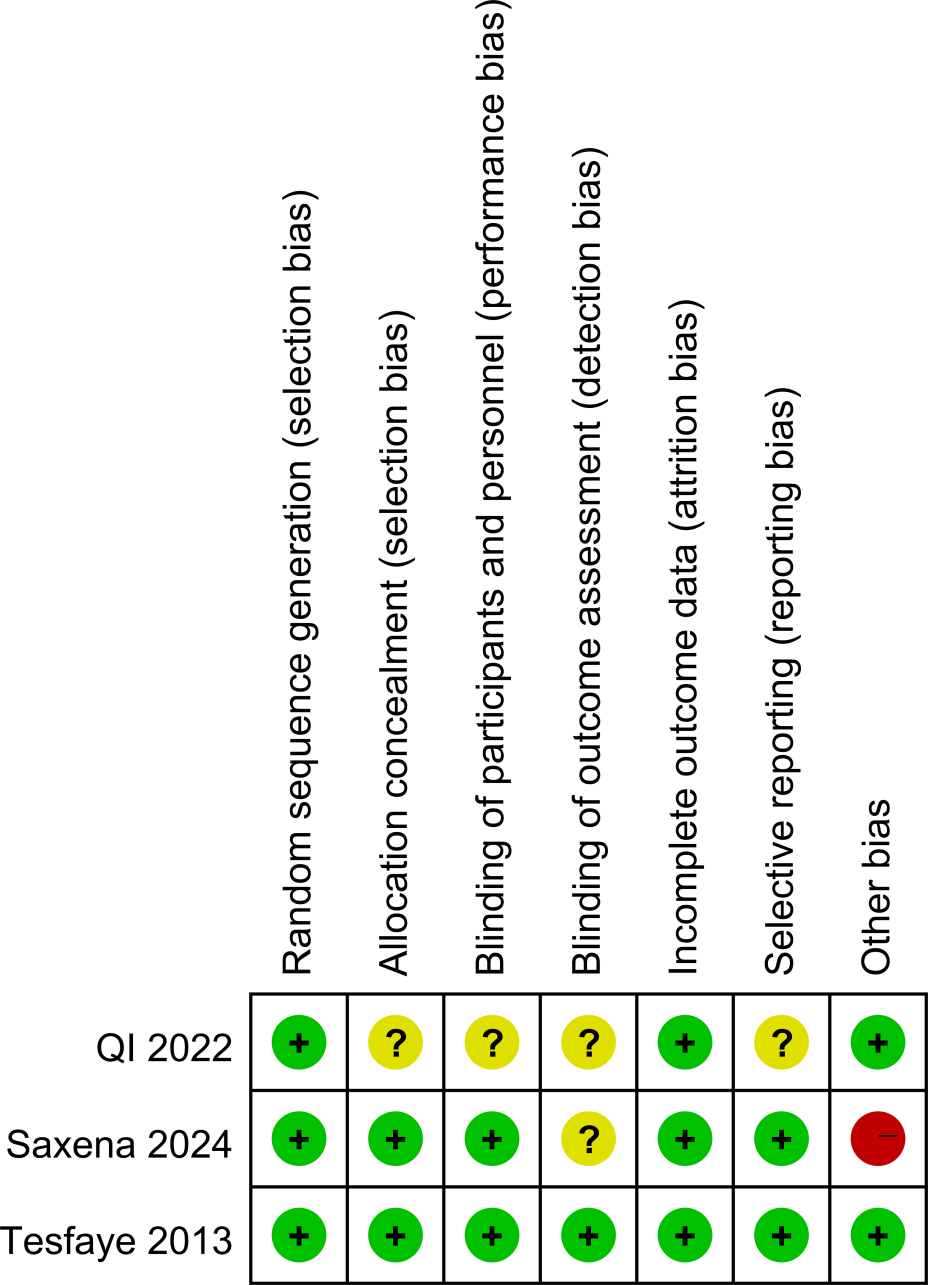
Quality evaluation of the included studies.

Risk of bias was assessed for each study. All three trials reported their methods for generating the random sequence (36–38). Two studies described the procedures used for allocation concealment, and two reported blinding of both investigators and participants (36, 37). In all three trials, outcome data were either complete or missing to an extent unlikely to affect the estimated treatment effects (36–38). These features supported a judgement of low risk of bias in most domains. However, one study had a very small sample size (n=30) (36), which raised concerns about other potential sources of bias and led to an overall rating of high risk for that trial. Details of the quality assessment are presented in **Figures 2**, **3**.

### 3.3 Efficacy analysis

A total of three randomized controlled trials were included. The primary outcome was short-term pain intensity assessed with the numerical rating scale (NRS). In two studies that could be pooled, combination therapy produced significantly greater pain reduction than monotherapy (MD=-1.82, 95%CI=-2.10, -1.54, *P*<0.00001). When analyses were stratified by the comparator drug, pregabalin plus duloxetine was more effective than pregabalin alone (MD=-1.53, 95%CI=-2.42, -0.64, *P* = 0.0008) than duloxetine alone (MD=-1.85, 95%CI=-2.14, -1.56, *P*<0.00001). The difference between these two subgroup effects was not statistically significant (*P* = 0.50).

For secondary continuous outcomes reported in single studies, all results were consistent with a benefit of combination therapy. Compared with monotherapy, the combination was associated with lower scores on the VAS (MD=-1.42, 95%CI=-1.83, -1.01, *P*<0.00001), BPI-MSF (MD=-1.46, 95%CI=-2.35, -0.57, *P* = 0.001), and PDQ (MD=-3.00, 95%CI=-5.55, -0.45, *P* = 0.02). Responder analyses showed that the proportion of patients achieving at least 50% pain reduction was higher with combination therapy than with duloxetine 120 mg alone (RR = 1.81, 95%CI=1.17, 2.81, *P* = 0.008), whereas no significant difference was observed when combination therapy was compared with pregabalin 600 mg (RR = 1.13, 95%CI=0.84, 1.50, *P* = 0.42). For the outcome of at least 30% pain reduction, differences between combination therapy and monotherapy were not statistically significant under either background treatment (combination versus pregabalin: RR = 1.04, 95%CI=0.83, 1.29, *P* = 0.75; combination versus duloxetine: RR = 1.24, 95%CI=0.91, 1.70, *P* = 0.17).

**Figure 4:**
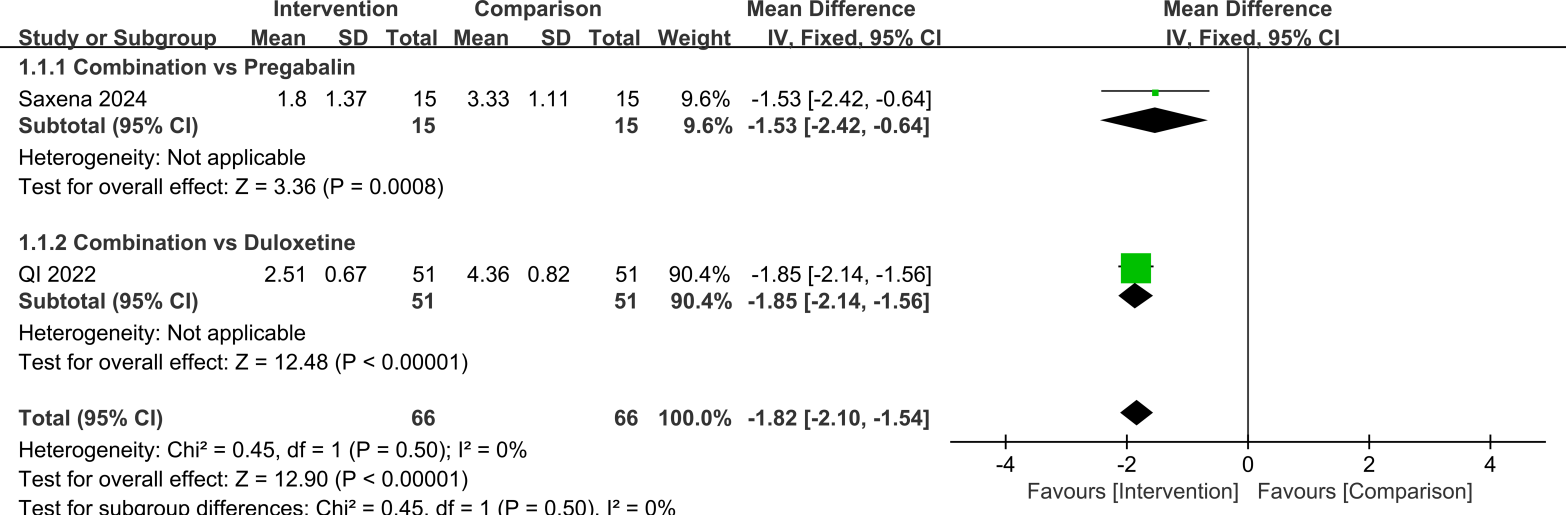
Comparison of NRS scores in PDN patients treated with pregabalin combined with duloxetine.

**Figure 5:**
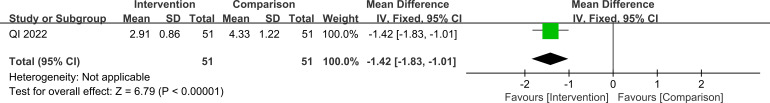
Comparison of VAS scores in PDN patients treated with pregabalin combined with duloxetine.

**Figure 6:**
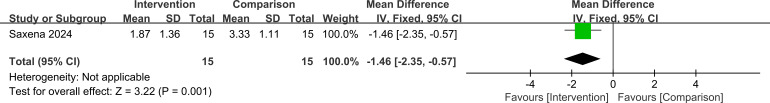
Comparison of BPI-MSF scores in PDN patients treated with pregabalin combined with duloxetine.

**Figure 7:**
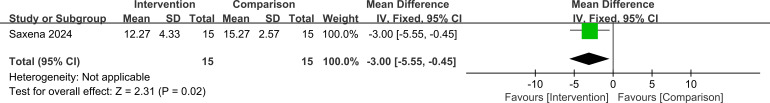
Comparison of PDQ scores in PDN patients treated with pregabalin combined with duloxetine.

**Figure 8:**
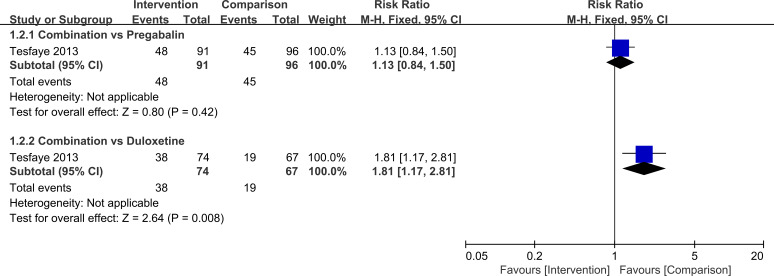
Comparison of the number of patients achieving≥50% pain relief in PDN patients treated with pregabalin combined with duloxetine.

**Figure 9:**
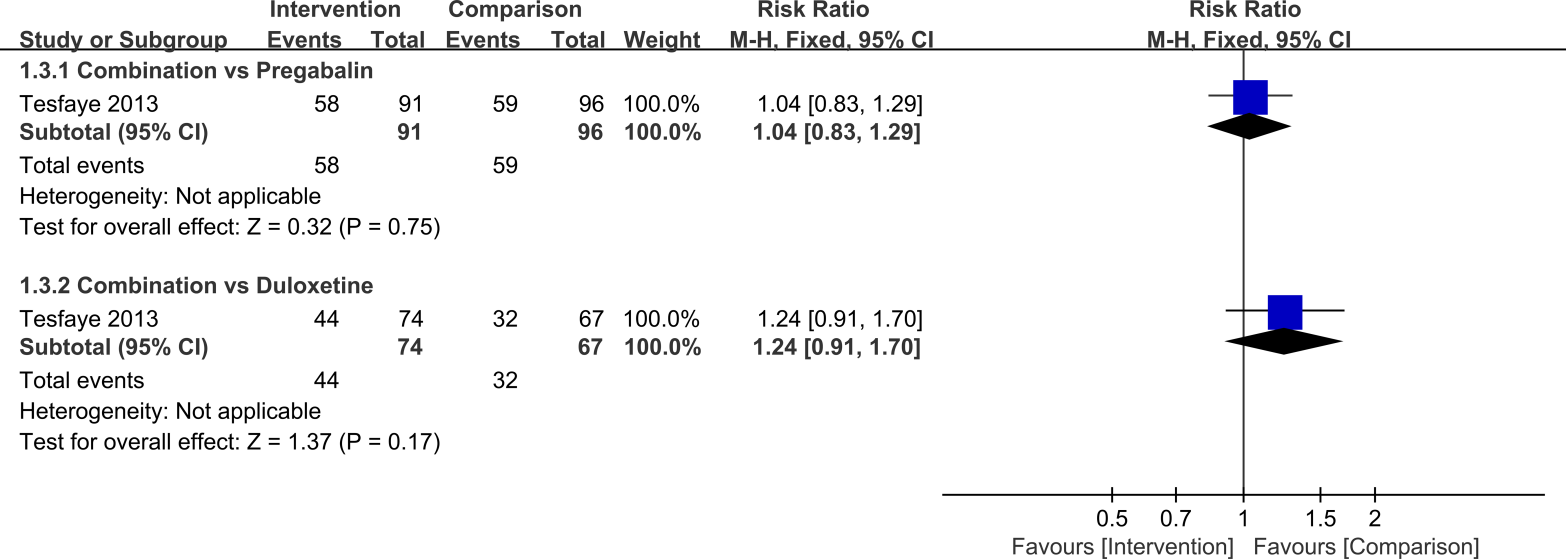
Comparison of the number of patients achieving≥30% pain relief in PDN patients treated with pregabalin combined with duloxetine.

Taken together, these findings suggest that combination therapy may reduce short-term pain intensity compared with monotherapy. However, the certainty of evidence for the pooled NRS outcome is low (**Supplementary Table 3**). This low certainty was driven in part by the inclusion of a small high-risk trial (*n* = 30) and the limited number of pooled studies. These results are illustrated in **Figures 4**–**9**.

### 3.4 Safety analysis

**Figure 10:**
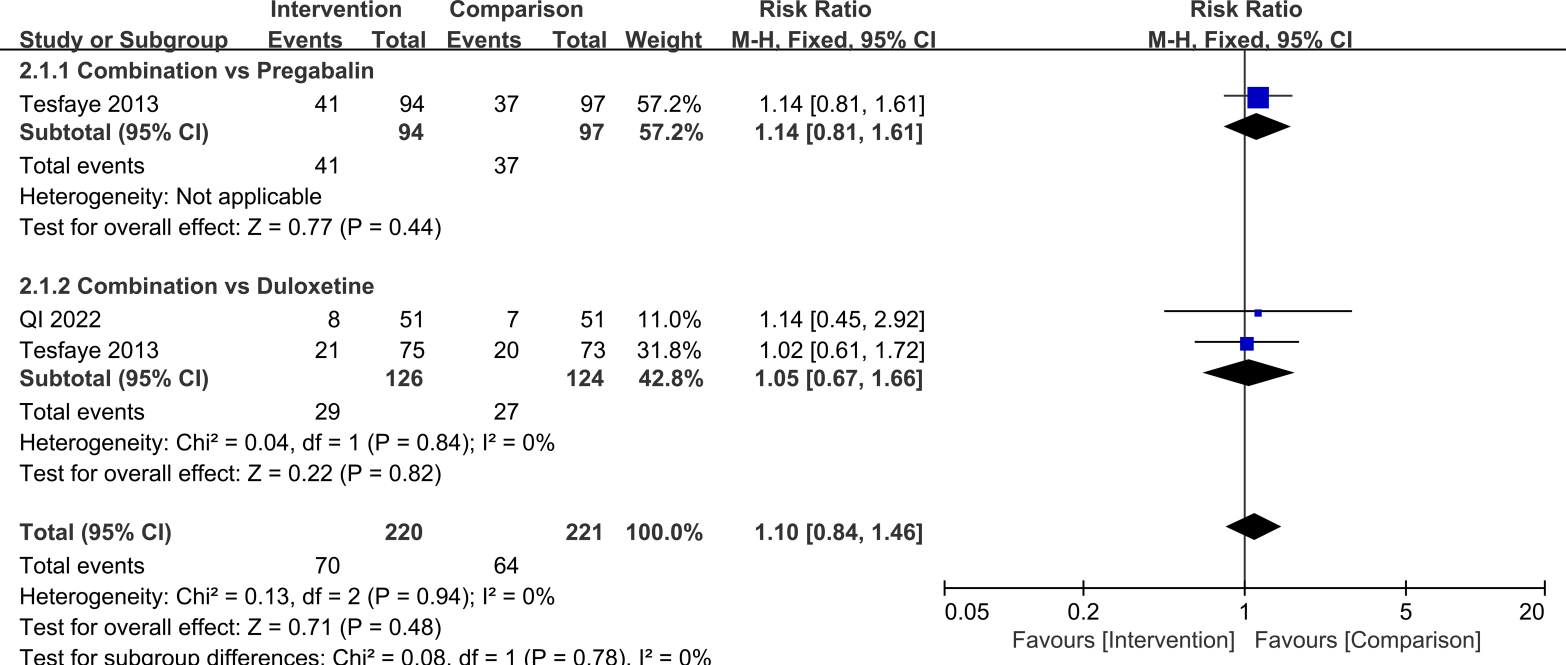
Comparison of adverse event incidence with pregabalin combined with duloxetine.

**Figure 11:**
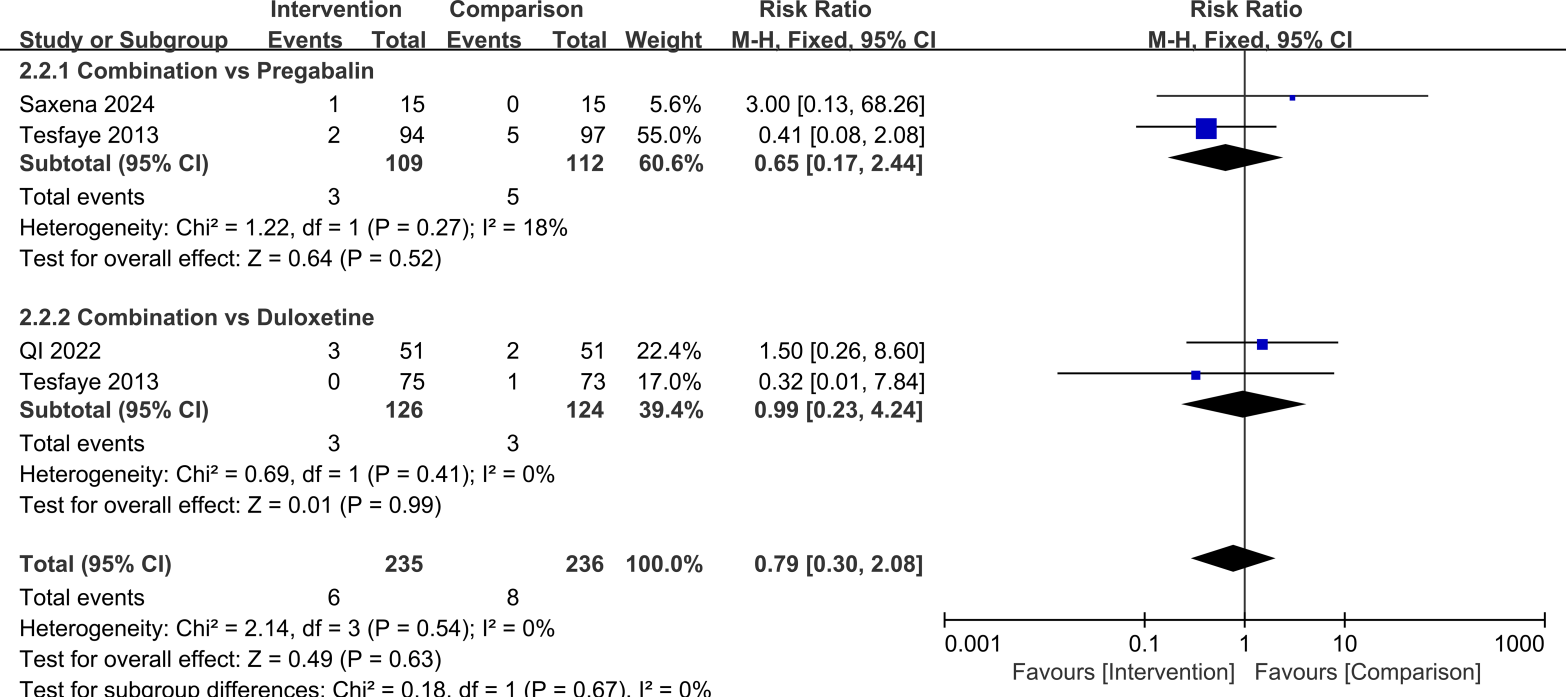
Comparison of somnolence incidence with pregabalin combined with duloxetine.

**Figure 12:**
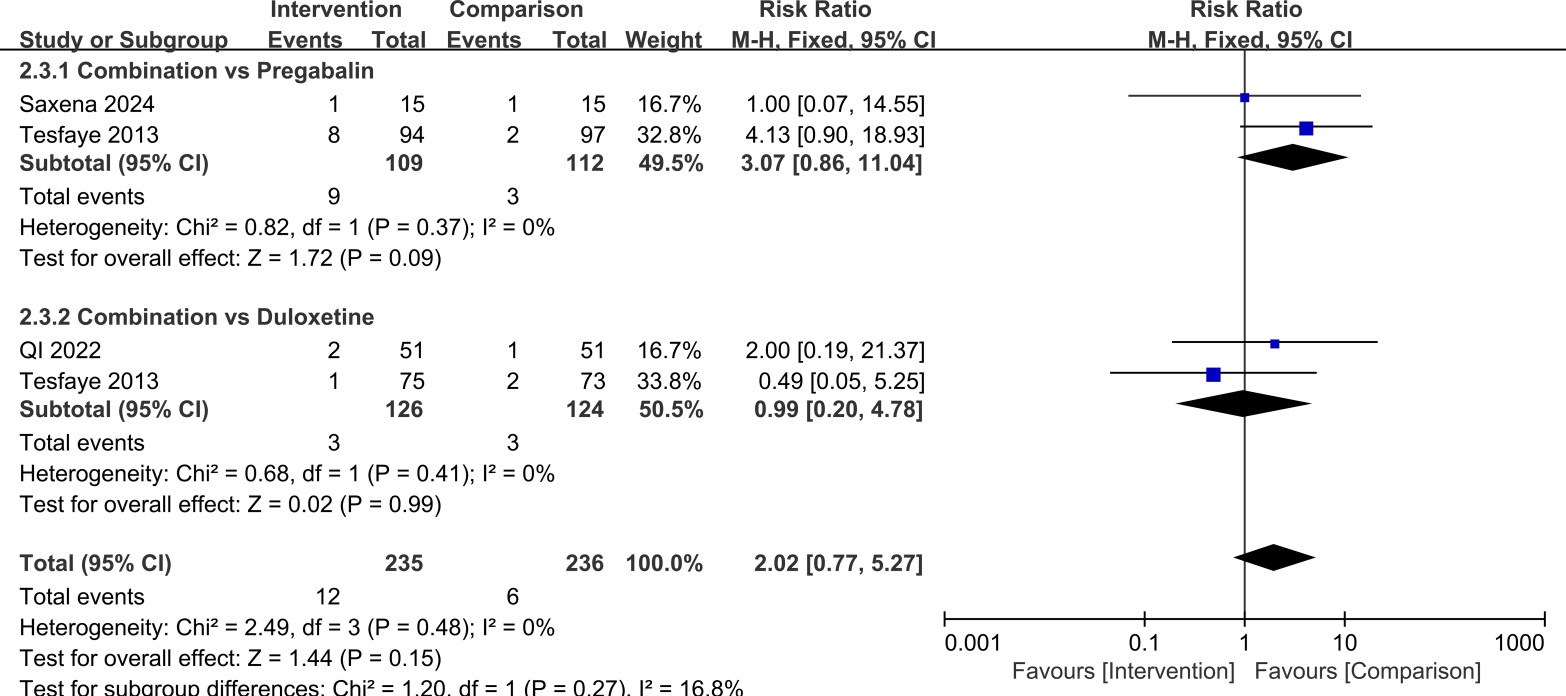
Comparison of nausea/vomiting incidence with pregabalin combined with duloxetine.

In terms of safety, there was no significant difference in the overall risk of adverse events or in the main specific adverse reactions between combination therapy and monotherapy. For any adverse event, RR = 1.10, 95%CI=0.84, 1.46, *P* = 0.48. In subgroup analyses by comparator drug, for combination therapy versus pregabalin alone, RR = 1.14, 95%CI=0.81, 1.61, *P* = 0.44 and for combination therapy versus duloxetine alone, RR = 1.05, 95%CI=0.67, 1.66, *P* = 0.82, with no significant difference between these subgroups (*P* = 0.78). For somnolence, the overall RR = 0.79, 95%CI=0.30, 2.08, *P* = 0.63, when stratified by control treatment, for combination therapy versus pregabalin, RR = 0.65, 95%CI=0.17, 2.44, *P* = 0.52 and for combination therapy versus duloxetine, RR = 0.99, 95%CI=0.23, 4.24, *P* = 0.99, with no significant difference between subgroups (*P* = 0.67). For nausea/vomiting, the overall RR = 2.02, 95%CI=0.77, 5.27, *P* = 0.15. In subgroup analyses, for combination therapy versus pregabalin, RR = 3.07, 95%CI=0.86, 11.04, *P* = 0.09 and for combination therapy versus duloxetine, RR = 0.99, 95%CI=0.20, 4.78, *P* = 0.99, with no significant difference between subgroups (*P* = 0.27). Taken together, these findings suggest that, based on the available evidence and current sample size, the overall tolerability of pregabalin plus duloxetine is comparable to that of single-drug therapy, and there is no clear signal of increased risk for common adverse events such as somnolence, nausea, or vomiting. However, the certainty of evidence for safety outcomes was low to very low because of few trials and very serious imprecision for specific adverse events (**Supplementary Table 3**). Larger, high-quality randomized trials are needed to confirm the safety profile of the combination regimen. These results are illustrated in **Figures 10**–**12**.

### 3.5 Results of certainty of evidence

Using the GRADE approach, we assessed the certainty of the evidence for each outcome. Because of concerns related to allocation concealment, small sample sizes, and marked differences in effect estimates when different comparator drugs were used within the same trial, the certainty of evidence for all outcomes was downgraded by at least one level. For some outcomes involving rare events, the certainty was downgraded by two levels due to serious imprecision. The detailed GRADE ratings for each outcome are presented in **Supplementary Table 3**.

### 3.6 Sensitivity analysis

We conducted sensitivity analyses by applying alternative statistical models. For efficacy outcomes, replacing the fixed-effect model with a random-effects model did not materially change the results. The *P* values remained highly significant for NRS (*P*<0.0001), VAS (*P*<0.0001), BPI-MSF (*P* = 0.001), and PDQ (*P* = 0.02), while the P values for ≥30% pain reduction (*P* = 0.27) and ≥50% pain reduction (*P* = 0.02) were also consistent with the primary analyses. For safety outcomes, changing from a fixed-effect to a random-effects model likewise did not alter the conclusions. For any adverse event, somnolence, and nausea or vomiting, the corresponding *P* values were 0.46, 0.67, and 0.22, respectively, which were very similar to the primary analyses. Overall, the findings were robust to the choice of statistical model (fixed- vs random-effects), with no material change in the direction or statistical significance of the results. However, the certainty of evidence remains low to very low (**Supplementary Table 3**) due to limited trial numbers and imprecision.

## 4 Discussion

This study compared the effectiveness and safety of pregabalin plus duloxetine versus pregabalin or duloxetine alone for painful diabetic neuropathy. Overall, the available evidence suggests that combination therapy may provide additional short-term pain relief compared with monotherapy. For secondary outcomes reported in single trials, results were generally consistent with a benefit of the combination regimen (VAS, BPI-MSF, PDQ, and responder outcomes), although the certainty for most of these outcomes was low to very low (**Supplementary Table 3**). The most frequently reported adverse effects were somnolence and nausea/vomiting. Across the included trials, no clear increase in overall adverse events or in these common adverse effects was observed with combination therapy. However, the certainty of evidence for safety outcomes remains low to very low because of few trials and imprecision.

Pregabalin acts mainly on peripheral nerves, it binds to the α2δ-1 subunit of voltage-gated calcium channels and reduces the release of several neurotransmitters, thereby dampening excessive excitation of peripheral neurons (39, 40). This mechanism helps to relieve pain that arises from damage to peripheral nerves. Duloxetine, by contrast, works primarily within the central nervous system. It inhibits the reuptake of serotonin (5-HT) and norepinephrine (NE), which enhances the activity of descending pain-inhibitory pathways (41). By strengthening these pathways, duloxetine increases the brain’s ability to suppress incoming pain signals and thus reduces the perception of pain. Because pregabalin and duloxetine act at different levels—pregabalin mainly in the periphery and duloxetine mainly in the central nervous system—their combined use may lead to an additive effect. Acting through two complementary pathways, the combination can simultaneously reduce the transmission and the perception of pain.

Several guidelines and clinical studies consistently identify pregabalin and duloxetine as first-line medicines for neuropathic pain in adults, including painful diabetic neuropathy (42–44). Placebo-controlled randomized trials have shown that both drugs provide a stable, moderate reduction in pain, but the proportion of patients who achieve a meaningful response is limited, and treatment discontinuation due to side effects is not uncommon. Reviews have further highlighted that although these drugs can clearly reduce pain, the need for dose titration and problems with tolerability often limit how far the dose of a single agent can be increased (25, 28). Randomized trials indicate that pregabalin at 300–600 mg per day can significantly reduce pain, but somnolence and dizziness occur frequently at these doses (45, 46). Duloxetine at 60–120 mg per day improves both pain and daily functioning, yet nausea, dry mouth, and other side effects may reduce adherence and make further dose escalation difficult (47–49). These data provide a real-world reference for the monotherapy arms in our analysis and help explain why simply increasing the dose of a single drug does not always move patients into the higher-response range, such as achieving at least 50% pain reduction.

Previous systematic reviews have noted that the overall evidence base for combination pharmacotherapy in neuropathic pain is limited. Existing studies vary widely in terms of drug pairings, dose selection and titration, and follow-up duration, which can contribute to clinical heterogeneity and inconsistent conclusions in pooled analyses (50). In contrast, our review focuses on a specific combination of pregabalin plus duloxetine and uses short-term pain intensity as the primary outcome, allowing pooling of two trials. Although the pooled estimate showed low statistical heterogeneity and a statistically significant effect, the overall certainty of evidence for the primary outcome remains low (**Supplementary Table 3**) because of the small number of trials and concerns related to risk of bias and imprecision.

Evidence from randomized controlled trials in other neuropathic or mixed pain conditions also suggests that combination therapy may be more effective than single-drug treatment (51). By acting on different pain pathways at the same time, combination regimens can produce synergistic or additive effects and thereby enhance pain relief. Similar findings have been reported in other types of neuropathic pain, such as postherpetic neuralgia and chronic back pain (51). In our analysis, the short-term reduction in pain intensity measured by NRS showed a pattern broadly consistent with these previous combination-therapy studies. Taken together, this external evidence provides biological plausibility for a potential additional benefit of pregabalin plus duloxetine in PDN. However, given the limited number of trials and low certainty of evidence, the magnitude of benefit should be interpreted cautiously.

Several studies suggest that a change of about 1–2 points on pain rating scales can be used as a reference for the minimal clinically important difference (MCID) in PDN (42, 43). In our review, the pooled short-term net change in NRS scores with combination therapy was approximately -1.5 to -2.0 points. This reduction is statistically significant and may meet or exceed commonly used MCID thresholds, suggesting potential additional pain relief compared with monotherapy. The magnitude of this effect is comparable with, and readily interpretable in the context of, the average treatment effects reported in key randomized trials of pregabalin or duloxetine monotherapy (45–49). However, the certainty of evidence for the pooled NRS outcome is low (**Supplementary Table 3**), reflecting the small number of trials and concerns related to risk of bias and imprecision. Furthermore, no clear increase in overall adverse events or in the most frequently reported side effects was observed with combination therapy, consistent with the known adverse-effect profiles of pregabalin and duloxetine (28, 49). However, because some adverse events were infrequent, the corresponding confidence intervals were wide, reflecting considerable imprecision; accordingly, the certainty of evidence for safety outcomes was low to very low. Larger, prospective trials are needed to provide more definitive reassurance regarding the safety of the combined regimen.

The findings of this review have potential implications for clinical practice. Based on the available randomized evidence, pregabalin combined with duloxetine may offer additional short-term pain relief compared with monotherapy for some patients with painful diabetic neuropathy. However, the certainty of evidence for the primary pain outcome is low and is limited by the small number of trials, including one small study at high risk of bias. Therefore, this regimen could be considered on an individualized basis, particularly for patients with an inadequate response to a single agent at usual doses, while taking comorbidities, tolerability, and patient preferences into account. Although no clear increase in common adverse events was observed, the certainty of evidence for safety outcomes remains low to very low and dosing and follow-up varied across trials, thus, routine broad adoption cannot be recommended without confirmation from larger, well-designed studies.

This study has several strengths. By focusing on a single fixed drug combination, it avoids the complexity and statistical heterogeneity associated with multiple different combination regimens and comparators. We also followed standardized analytic procedures using RevMan and applied the GRADE framework to rate the certainty of evidence. Heterogeneity, subgroup differences, and certainty judgments were explicitly reported. For the primary pain outcome, pooling was feasible for two trials, and statistical heterogeneity was low. However, given the limited number of trials, low statistical heterogeneity does not necessarily imply high certainty of evidence. Several important limitations should be noted. The number of available trials was small, and many outcomes were derived from single studies or involved rare events, leading to imprecision and a potential risk of publication bias. One included trial (Saxena 2024, n=30) was rated at high risk of bias. Although its statistical weight in the pooled NRS analysis was small and the pooled estimate was largely driven by the larger trial, the inclusion of a high-risk small study and the limited number of trials reduce confidence in the robustness of the findings. Because only two trials contributed to the pooled NRS outcome, excluding one trial would leave a single-study estimate and is therefore of limited interpretability; accordingly, we assessed influence qualitatively based on study weights and risk-of-bias ratings. In addition, clinical heterogeneity across trials may limit generalizability, including variability in dosing (pregabalin approximately 150–600 mg/day; duloxetine approximately 60–120 mg/day) and follow-up duration (4–16 weeks). Blinding and allocation concealment were not always fully reported, which may have introduced bias, especially for subjective outcomes such as pain scores. These limitations should be taken into account when interpreting our findings.

Future studies should include adequately powered, double-blind, multicenter randomized controlled trials with standardized dosing and titration protocols, a unified follow-up window, and a core set of outcomes. Such trials are needed to determine the most effective dosing regimens and treatment durations, to explore dose–response relationships and individualized dosing strategies, and to extend follow-up long enough to assess the durability of symptom relief and long-term safety. In addition, mechanistic studies would be valuable to clarify how pregabalin and duloxetine interact to produce combined analgesic effects.

## 5 Conclusion

Low-certainty evidence suggests that pregabalin combined with duloxetine may improve short-term pain intensity compared with monotherapy in painful diabetic neuropathy. Evidence for responder outcomes and safety is limited, with low to very low certainty for most outcomes due to risk of bias and imprecision. Larger, adequately powered, double-blind trials with standardized dosing and longer follow-up are needed to confirm both efficacy and safety.

## Data Availability

The original contributions presented in the study are included in the article/**Supplementary Material**. Further inquiries can be directed to the corresponding author.
