# Supplementary material for: Pregabalin and duloxetine combination for painful diabetic neuropathy: a systematic review and meta-analysis
Source: Front Endocrinol (Lausanne). 2026 Mar 11;17:1750441. doi: 10.3389/fendo.2026.1750441 (PMC13012920; doi:10.3389/fendo.2026.1750441)
Supplement: Supplementary file 2 [file Table2.docx]

| Pubmed | |
| --- | --- |
| #1 | (((((((((((("Pregabalin"[Mesh]) OR ((R-)-3-isobutyl GABA[Title/Abstract])) OR ((S+)-3-isobutyl GABA[Title/Abstract])) OR (3-isobutyl GABA[Title/Abstract])) OR (3 isobutyl GABA[Title/Abstract])) OR (GABA, 3-isobutyl[Title/Abstract])) OR (3-(aminomethyl)-5-methylhexanoic acid[Title/Abstract])) OR ((S)-3-(aminomethyl)-5-methylhexanoic acid[Title/Abstract])) OR (CI 1008[Title/Abstract])) OR (1008, CI[Title/Abstract])) OR (CI-1008[Title/Abstract])) OR (CI1008[Title/Abstract])) OR (Lyrica[Title/Abstract]) |
| #2 | ((((((((((((((("Duloxetine Hydrochloride"[Mesh]) OR (Hydrochloride, Duloxetine[Title/Abstract])) OR (Duloxetine HCl[Title/Abstract])) OR (HCl, Duloxetine[Title/Abstract])) OR (Duloxetine[Title/Abstract])) OR (N-methyl-3-(1-naphthalenyloxy)-2-thiophenepropanamine[Title/Abstract])) OR (N-methyl-3-(1-naphthalenyloxy)-3-(2-thiophene)propanamide[Title/Abstract])) OR (LY 227942[Title/Abstract])) OR (LY-227942[Title/Abstract])) OR (LY227942[Title/Abstract])) OR (LY 248686[Title/Abstract])) OR (LY-248686[Title/Abstract])) OR (LY248686[Title/Abstract])) OR (Duloxetine Ethanedioate (1:1), (+-)-isomer - T353987[Title/Abstract])) OR (Cymbalta[Title/Abstract])) OR (Duloxetine, (+)-isomer[Title/Abstract]) |
| #3 | ((((((((((((((((((((((((((((((((((((((((((((("Diabetic Neuropathies"[Mesh]) OR (Diabetic Neuropathy[Title/Abstract])) OR (Neuropathies, Diabetic[Title/Abstract])) OR (Neuropathy, Diabetic[Title/Abstract])) OR (Diabetic Amyotrophy[Title/Abstract])) OR (Amyotrophies, Diabetic[Title/Abstract])) OR (Amyotrophy, Diabetic[Title/Abstract])) OR (Diabetic Amyotrophies[Title/Abstract])) OR (Diabetic Autonomic Neuropathy[Title/Abstract])) OR (Autonomic Neuropathies, Diabetic[Title/Abstract])) OR (Autonomic Neuropathy, Diabetic[Title/Abstract])) OR (Diabetic Autonomic Neuropathies[Title/Abstract])) OR (Neuropathies, Diabetic Autonomic[Title/Abstract])) OR (Neuropathy, Diabetic Autonomic[Title/Abstract])) OR (Diabetic Neuralgia[Title/Abstract])) OR (Diabetic Neuralgias[Title/Abstract])) OR (Neuralgias, Diabetic[Title/Abstract])) OR (Neuralgia, Diabetic[Title/Abstract])) OR (Diabetic Neuropathy, Painful[Title/Abstract])) OR (Diabetic Neuropathies, Painful[Title/Abstract])) OR (Neuropathies, Painful Diabetic[Title/Abstract])) OR (Neuropathy, Painful Diabetic[Title/Abstract])) OR (Painful Diabetic Neuropathies[Title/Abstract])) OR (Painful Diabetic Neuropathy[Title/Abstract])) OR (Diabetic Polyneuropathy[Title/Abstract])) OR (Diabetic Polyneuropathies[Title/Abstract])) OR (Polyneuropathies, Diabetic[Title/Abstract])) OR (Polyneuropathy, Diabetic[Title/Abstract])) OR (Asymmetric Diabetic Proximal Motor Neuropathy[Title/Abstract])) OR (Diabetic Asymmetric Polyneuropathy[Title/Abstract])) OR (Asymmetric Polyneuropathies, Diabetic[Title/Abstract])) OR (Asymmetric Polyneuropathy, Diabetic[Title/Abstract])) OR (Diabetic Asymmetric Polyneuropathies[Title/Abstract])) OR (Polyneuropathies, Diabetic Asymmetric[Title/Abstract])) OR (Polyneuropathy, Diabetic Asymmetric[Title/Abstract])) OR (Diabetic Mononeuropathy[Title/Abstract])) OR (Diabetic Mononeuropathies[Title/Abstract])) OR (Mononeuropathies, Diabetic[Title/Abstract])) OR (Diabetic Mononeuropathy Simplex[Title/Abstract])) OR (Diabetic Mononeuropathy Simplices[Title/Abstract])) OR (Mononeuropathy Simplex, Diabetic[Title/Abstract])) OR (Mononeuropathy Simplices, Diabetic[Title/Abstract])) OR (Simplex, Diabetic Mononeuropathy[Title/Abstract])) OR (Simplices, Diabetic Mononeuropathy[Title/Abstract])) OR (Mononeuropathy, Diabetic[Title/Abstract])) OR (Symmetric Diabetic Proximal Motor Neuropathy[Title/Abstract]) |
| #4 | (("Randomized Controlled Trials as Topic"[Mesh]) OR ("Randomized Controlled Trial" [Publication Type])) OR (("Clinical Trial" [Publication Type]) OR ("Clinical Trials as Topic"[Mesh])) |
| #5 | #1 AND #2 AND #3 AND #4 |

| Embase | |
| --- | --- |
| #1 | 'pregabalin'/exp OR 'pregabalin' OR '3 aminomethyl 5 methylhexanoic acid':ab,ti OR '3 isobutyl 4 aminobutanoic acid':ab,ti OR '3 isobutyl 4 aminobutyric acid':ab,ti OR '3 isobutyl gaba':ab,ti OR '3 isobutylgaba':ab,ti OR '4 amino 3 (2 methylpropyl) butanoic acid':ab,ti OR '4 amino 3 isobutylbutanoic acid':ab,ti OR '4 amino 3 isobutylbutyric acid':ab,ti OR 'algerika':ab,ti OR 'alivax (pregabalin)':ab,ti OR 'alyse (drug)':ab,ti OR 'alzain':ab,ti OR 'andogablin':ab,ti OR 'aprion (drug)':ab,ti OR 'averopreg':ab,ti OR 'axalid':ab,ti OR 'axual':ab,ti OR 'balfibro':ab,ti OR 'bonqat':ab,ti OR 'brieka':ab,ti OR 'ci 1008':ab,ti OR 'ci1008':ab,ti OR 'clasica (drug)':ab,ti OR 'convugabalin':ab,ti OR 'dismedox':ab,ti OR 'dolica':ab,ti OR 'dragonor':ab,ti OR 'ecubalin':ab,ti OR 'epica':ab,ti OR 'epiron':ab,ti OR 'gaba-p':ab,ti OR 'gabanext':ab,ti OR 'gabarol':ab,ti OR 'gabarol cr':ab,ti OR 'gabica':ab,ti OR 'gablin':ab,ti OR 'gablovac':ab,ti OR 'gabrika':ab,ti OR 'gavin (pregabalin)':ab,ti OR 'gialtyn':ab,ti OR 'glonervya':ab,ti OR 'helimon':ab,ti OR 'hexgabalin':ab,ti OR 'irenypathic':ab,ti OR 'kabian (pregabalin)':ab,ti OR 'kemirica':ab,ti OR 'kineptia (pregabalin)':ab,ti OR 'lecaent':ab,ti OR 'lingabat':ab,ti OR 'linprel':ab,ti OR 'lyribastad':ab,ti OR 'lyric':ab,ti OR 'lyrica':ab,ti OR 'lyrica cr':ab,ti OR 'lyrineur':ab,ti OR 'lyrolin':ab,ti OR 'lyzalon':ab,ti OR 'martesia (drug)':ab,ti OR 'maxgalin':ab,ti OR 'maxgalin er':ab,ti OR 'misabri':ab,ti OR 'misabri pr':ab,ti OR 'mystika':ab,ti OR 'nervalin':ab,ti OR 'nervalin cr':ab,ti OR 'neugaba':ab,ti OR 'neugaba er':ab,ti OR 'neuragabalin':ab,ti OR 'neurega':ab,ti OR 'neurica':ab,ti OR 'neuristan':ab,ti OR 'neurolin':ab,ti OR 'neurovan':ab,ti OR 'neurum':ab,ti OR 'nuramed':ab,ti OR 'nurogab':ab,ti OR 'paden':ab,ti OR 'pagadin':ab,ti OR 'pagamax':ab,ti OR 'painica':ab,ti OR 'pd 144723':ab,ti OR 'pd144723':ab,ti OR 'pergadel':ab,ti OR 'plenica':ab,ti OR 'pragiola':ab,ti OR 'prebanal':ab,ti OR 'prebel':ab,ti OR 'prebictal':ab,ti OR 'prebien':ab,ti OR 'prefaxil (pregabalin)':ab,ti OR 'pregalin':ab,ti OR 'pregalin sr':ab,ti OR 'pregalodos':ab,ti OR 'pregamid':ab,ti OR 'pregan':ab,ti OR 'pregastar':ab,ti OR 'pregatrend':ab,ti OR 'pregavalex':ab,ti OR 'pregeb':ab,ti OR 'pregeb od':ab,ti OR 'pregobin':ab,ti OR 'prelin':ab,ti OR 'priga':ab,ti OR 'provelyn':ab,ti OR 'regapen':ab,ti OR 'symra':ab,ti OR 'vronogabic':ab,ti OR 'xablin':ab,ti OR 'xil':ab,ti OR 'ynp 1807':ab,ti OR 'ynp1807':ab,ti OR 'pregabalin':ab,ti |
| #2 | 'duloxetine'/exp OR '3 (1 naphthyloxy) 3 (2 thienyl) n methylpropylamine':ab,ti OR '3 (naphth 1 yloxy) 3 (thien 2 yl) n methylpropylamine':ab,ti OR 'ariclaim':ab,ti OR 'cymbalta':ab,ti OR 'dlx iso3':ab,ti OR 'dlxiso3':ab,ti OR 'drizalma':ab,ti OR 'drizalma sprinkle':ab,ti OR 'dulane':ab,ti OR 'duloxetine boehringer ingelheim':ab,ti OR 'duloxetine hydrochloride':ab,ti OR 'duzela':ab,ti OR 'ly 248686':ab,ti OR 'ly248686':ab,ti OR 'n methyl 3 (1 naphthalenyloxy) 2 thiophenepropanamine':ab,ti OR 'n methyl 3 (1 naphthalenyloxy) 3 (2 thiophenyl) 1 propanamine':ab,ti OR 'n methyl 3 (1 naphthyloxy) 3 (2 thienyl) propylamine':ab,ti OR 'n methyl 3 (naphth 1 yloxy) 3 (thien 2 yl) propylamine':ab,ti OR 'n methyl 3 (naphthalen 1 yloxy) 2 thiophenepropanamine':ab,ti OR 'n methyl 3 (naphthalen 1 yloxy) 3 (thiophen 2 yl) propan 1 amine':ab,ti OR 'nodetrip':ab,ti OR 'xeristar':ab,ti OR 'yentreve':ab,ti OR 'duloxetine':ab,ti |
| #3 | 'diabetic neuropathy'/exp OR 'diabetes mellitus with neuropathy':ab,ti OR 'diabetes neuropathy':ab,ti OR 'diabetic mononeuritis':ab,ti OR 'diabetic mononeuropathy':ab,ti OR 'diabetic neuritis':ab,ti OR 'diabetic neuropathies':ab,ti OR 'diabetic peripheral neuropathy':ab,ti OR 'diabetic peripheral polyneuropathy':ab,ti OR 'diabetic polyneuritis':ab,ti OR 'diabetic polyneuropathy':ab,ti OR 'diabetic sensorimotor polyneuropathy':ab,ti OR 'neuropathies in diabetes':ab,ti OR 'neuropathy in diabetes':ab,ti OR 'peripheral diabetic neuropathy':ab,ti OR 'polyneuropathy in diabetes':ab,ti OR 'diabetic neuropathy':ab,ti |
| #4 | 'randomized controlled trial'/exp OR 'randomized controlled trial (topic)'/exp OR 'controlled trial, randomized':ab,ti OR 'randomised controlled study':ab,ti OR 'randomised controlled trial':ab,ti OR 'randomized controlled study':ab,ti OR 'trial, randomized controlled':ab,ti OR 'randomized controlled trial':ab,ti OR 'pragmatic clinical trials as topic':ab,ti OR 'randomized controlled trials':ab,ti OR 'randomized controlled trials as topic':ab,ti OR 'randomized controlled trial (topic)':ab,ti OR 'clinical trial'/exp OR 'clinical trial (topic)'/exp OR 'clinical drug trial':ab,ti OR 'major clinical trial':ab,ti OR 'trial, clinical':ab,ti OR 'clinical trial':ab,ti OR 'clinical trials':ab,ti OR 'clinical trials as topic':ab,ti OR 'clinical trial (topic)':ab,ti |
| #5 | #1 AND #2 AND #3 AND #4 |

| Web of Science | |
| --- | --- |
| #1 | “Pregabalin” OR “(R-)-3-isobutyl GABA” OR “(S+)-3-isobutyl GABA” OR “3-isobutyl GABA” OR “3 isobutyl GABA” OR “GABA, 3-isobutyl” OR “3-(aminomethyl)-5-methylhexanoic acid” OR “(S)-3-(aminomethyl)-5-methylhexanoic acid” OR “CI 1008” OR “1008, CI” OR “CI-1008” OR “CI1008” OR “Lyrica” |
| #2 | “Duloxetine Hydrochloride” OR “Hydrochloride, Duloxetine” OR “Duloxetine HCl” OR “HCl, Duloxetine” OR “Duloxetine” OR “N-methyl-3-(1-naphthalenyloxy)-2-thiophenepropanamine” OR “N-methyl-3-(1-naphthalenyloxy)-3-(2-thiophene)propanamide” OR “LY 227942” OR “LY-227942” OR “LY227942” OR “LY 248686” OR “LY-248686” OR “LY248686” OR “Duloxetine Ethanedioate (1:1), (+-)-isomer - T353987” OR “Cymbalta” OR “Duloxetine, (+)-isomer” |
| #3 | “Diabetic Neuropathies” OR “Diabetic Neuropathy” OR “Neuropathies, Diabetic” OR “Neuropathy, Diabetic” OR “Diabetic Amyotrophy” OR “Amyotrophies, Diabetic” OR “Amyotrophy, Diabetic” OR “Diabetic Amyotrophies” OR “Diabetic Autonomic Neuropathy” OR “Autonomic Neuropathies, Diabetic” OR “Autonomic Neuropathy, Diabetic” OR “Diabetic Autonomic Neuropathies” OR “Neuropathies, Diabetic Autonomic” OR “Neuropathy, Diabetic Autonomic” OR “Diabetic Neuralgia” OR “Diabetic Neuralgias” OR “Neuralgias, Diabetic” OR “Neuralgia, Diabetic” OR “Diabetic Neuropathy, Painful” OR “Diabetic Neuropathies, Painful” OR “Neuropathies, Painful Diabetic” OR “Neuropathy, Painful Diabetic” OR “Painful Diabetic Neuropathies” OR “Painful Diabetic Neuropathy” OR “Diabetic Polyneuropathy” OR “Diabetic Polyneuropathies” OR “Polyneuropathies, Diabetic” OR “Polyneuropathy, Diabetic” OR “Asymmetric Diabetic Proximal Motor Neuropathy” OR “Diabetic Asymmetric Polyneuropathy” OR “Asymmetric Polyneuropathies, Diabetic” OR “Asymmetric Polyneuropathy, Diabetic” OR “Diabetic Asymmetric Polyneuropathies” OR “Polyneuropathies, Diabetic Asymmetric” OR “Polyneuropathy, Diabetic Asymmetric” OR “Diabetic Mononeuropathy” OR “Diabetic Mononeuropathies” OR “Mononeuropathies, Diabetic” OR “Diabetic Mononeuropathy Simplex” OR “Diabetic Mononeuropathy Simplices” OR “Mononeuropathy Simplex, Diabetic” OR “Mononeuropathy Simplices, Diabetic” OR “Simplex, Diabetic Mononeuropathy” OR “Simplices, Diabetic Mononeuropathy” OR “Mononeuropathy, Diabetic” OR “Symmetric Diabetic Proximal Motor Neuropathy” |
| #4 | “Randomized Controlled Trial” OR “Randomized Controlled Trial* as Topic” OR “Clinical Trial*, Randomized” OR “Trial*, Randomized Clinical” OR “Controlled Clinical Trial*, Randomized” OR “Clinical Trial*” OR “trial, clinical” OR “clinical drug trial” OR “Trial*” OR “Clinical Trial* as Topic” |
| #5 | #1 AND #2 AND #3 AND #4 |

| The Cochrane Library | |
| --- | --- |
| #1 | MeSH descriptor: [Pregabalin] explode all trees |
| #2 | ((S) 3 isobutyl GABA):ti,ab,kw OR (GABA, 3 isobutyl):ti,ab,kw OR ((S) 3 (aminomethyl) 5 methylhexanoic acid):ti,ab,kw OR (3 isobutyl GABA):ti,ab,kw OR (3 (aminomethyl) 5 methylhexanoic acid):ti,ab,kw OR (3 isobutyl GABA):ti,ab,kw OR ((R ) 3 isobutyl GABA):ti,ab,kw OR (Lyrica):ti,ab,kw OR (CI 1008):ti,ab,kw OR (1008, CI):ti,ab,kw OR (CI1008):ti,ab,kw OR (CI 1008):ti,ab,kw |
| #3 | #1 OR #2 |
| #4 | MeSH descriptor: [Duloxetine Hydrochloride] explode all trees |
| #5 | (Hydrochloride, Duloxetine):ti,ab,kw OR (Duloxetine HCl):ti,ab,kw OR (HCl, Duloxetine):ti,ab,kw OR (Duloxetine):ti,ab,kw OR (N methyl 3 (1 naphthalenyloxy) 2 thiophenepropanamine):ti,ab,kw OR (N methyl 3 (1 naphthalenyloxy) 3 (2 thiophene)propanamide):ti,ab,kw OR (LY 227942):ti,ab,kw OR (LY 227942):ti,ab,kw OR (LY227942):ti,ab,kw OR (LY 248686):ti,ab,kw OR (LY 248686):ti,ab,kw OR (LY248686):ti,ab,kw OR (Duloxetine Ethanedioate (1 1), isomer T353987):ti,ab,kw OR (Cymbalta):ti,ab,kw OR (Duloxetine, isomer):ti,ab,kw |
| #6 | #4 OR #5 |
| #7 | MeSH descriptor: [Diabetic Neuropathies] explode all trees |
| #8 | (Diabetic Neuropathy):ti,ab,kw OR (Neuropathies, Diabetic):ti,ab,kw OR (Neuropathy, Diabetic):ti,ab,kw OR (Diabetic Amyotrophy):ti,ab,kw OR (Amyotrophies, Diabetic):ti,ab,kw OR (Amyotrophy, Diabetic):ti,ab,kw OR (Diabetic Amyotrophies):ti,ab,kw OR (Diabetic Autonomic Neuropathy):ti,ab,kw OR (Autonomic Neuropathies, Diabetic):ti,ab,kw OR (Autonomic Neuropathy, Diabetic):ti,ab,kw OR (Diabetic Autonomic Neuropathies):ti,ab,kw OR (Neuropathies, Diabetic Autonomic):ti,ab,kw OR (Neuropathy, Diabetic Autonomic):ti,ab,kw OR (Diabetic Neuralgia):ti,ab,kw OR (Diabetic Neuralgias):ti,ab,kw OR (Neuralgias, Diabetic):ti,ab,kw OR (Neuralgia, Diabetic):ti,ab,kw OR (Diabetic Neuropathy, Painful):ti,ab,kw OR (Diabetic Neuropathies, Painful):ti,ab,kw OR (Neuropathies, Painful Diabetic):ti,ab,kw OR (Neuropathy, Painful Diabetic):ti,ab,kw OR (Painful Diabetic Neuropathies):ti,ab,kw OR (Painful Diabetic Neuropathy):ti,ab,kw OR (Diabetic Polyneuropathy):ti,ab,kw OR (Diabetic Polyneuropathies):ti,ab,kw OR (Polyneuropathies, Diabetic):ti,ab,kw OR (Polyneuropathy, Diabetic):ti,ab,kw OR (Asymmetric Diabetic Proximal Motor Neuropathy):ti,ab,kw OR (Diabetic Asymmetric Polyneuropathy):ti,ab,kw OR (Asymmetric Polyneuropathies, Diabetic):ti,ab,kw OR (Asymmetric Polyneuropathy, Diabetic):ti,ab,kw OR (Diabetic Asymmetric Polyneuropathies):ti,ab,kw OR (Polyneuropathies, Diabetic Asymmetric):ti,ab,kw OR (Polyneuropathy, Diabetic Asymmetric):ti,ab,kw OR (Diabetic Mononeuropathy):ti,ab,kw OR (Diabetic Mononeuropathies):ti,ab,kw OR (Mononeuropathies, Diabetic):ti,ab,kw OR (Diabetic Mononeuropathy Simplex):ti,ab,kw OR (Diabetic Mononeuropathy Simplices):ti,ab,kw OR (Mononeuropathy Simplex, Diabetic):ti,ab,kw OR (Mononeuropathy Simplices, Diabetic):ti,ab,kw OR (Simplex, Diabetic Mononeuropathy):ti,ab,kw OR (Simplices, Diabetic Mononeuropathy):ti,ab,kw OR (Mononeuropathy, Diabetic):ti,ab,kw OR (Symmetric Diabetic Proximal Motor Neuropathy):ti,ab,kw |
| #9 | #7 OR #8 |
| #13 | #3 AND #6 AND #9 in Trials |

| 知网 |
| --- |
| （主题：普瑞巴林）OR（篇关摘：(S)-3-(氨甲基)-5-甲基己 + 3-异丁基GABA + (R-)-3-异丁基GABA + (S+)-3-异丁基GABA + 利痛抑 + CI 1008 + CI-1008(精确)）AND（主题：盐酸度洛西汀）OR（篇关摘：LY-227942 + N-甲基-3- + N-甲基-3- + 度洛西汀 + 欣百达 + 盐酸度洛西丁 + LY 248686 + LY-248686 + 度洛西汀乙二酸 + LY 227942 + LY227942(精确)）AND（主题：糖尿病神经病变）OR（篇关摘：糖尿病神经痛 + 对称性糖尿病性近端运动神经病 + 非对称性糖尿病性近端运动神经病 + 糖尿病不对称性多神经病 + 糖尿病单一神经病变 + 单纯糖尿病性单神经病 + 糖尿病自主神经病变(精确)）OR（篇关摘：疼痛性糖尿病性神经病 + 糖尿病肌萎缩 + 糖尿病多发神经病变(精确)） |

| 万方 |
| --- |
| 主题:(普瑞巴林 OR (S)-3-(氨甲基)-5-甲基己 OR 3-异丁基GABA OR (R-)-3-异丁基GABA OR (S+)-3-异丁基GABA OR 利痛抑 OR CI 1008 OR CI-1008) and 主题:(盐酸度洛西汀 OR LY-227942 OR N-甲基-3- OR N-甲基-3- OR 度洛西汀 OR 欣百达 OR 盐酸度洛西丁 OR LY 248686 OR LY-248686 OR 度洛西汀乙二酸 OR LY 227942 OR LY227942) and 主题:(糖尿病神经病变 OR 糖尿病神经痛 OR 对称性糖尿病性近端运动神经病 OR 非对称性糖尿病性近端运动神经病 OR 糖尿病不对称性多神经病 OR 糖尿病单一神经病变 OR 单纯糖尿病性单神经病 OR 糖尿病自主神经病变 OR 疼痛性糖尿病性神经病 OR 糖尿病肌萎缩 OR 糖尿病多发神经病变) |

| 维普 |
| --- |
| 题名或关键词=(普瑞巴林 OR (S)-3-(氨甲基)-5-甲基己 OR 3-异丁基GABA OR (R-)-3-异丁基GABA OR (S+)-3-异丁基GABA OR 利痛抑 OR CI 1008 OR CI-1008) 与 题名或关键词=(盐酸度洛西汀 OR LY-227942 OR N-甲基-3- OR N-甲基-3- OR 度洛西汀 OR 欣百达 OR 盐酸度洛西丁 OR LY 248686 OR LY-248686 OR 度洛西汀乙二酸 OR LY 227942 OR LY227942) 与 题名或关键词=(糖尿病神经病变 OR 糖尿病神经痛 OR 对称性糖尿病性近端运动神经病 OR 非对称性糖尿病性近端运动神经病 OR 糖尿病不对称性多神经病 OR 糖尿病单一神经病变 OR 单纯糖尿病性单神经病 OR 糖尿病自主神经病变 OR 疼痛性糖尿病性神经病 OR 糖尿病肌萎缩 OR 糖尿病多发神经病变) |
