# Supplementary material for: Pregabalin and duloxetine combination for painful diabetic neuropathy: a systematic review and meta-analysis
Source: Front Endocrinol (Lausanne). 2026 Mar 11;17:1750441. doi: 10.3389/fendo.2026.1750441 (PMC13012920; doi:10.3389/fendo.2026.1750441)
Supplement: Supplementary file 3 [file Table3.docx]

| Outcomes | Included studies | Number of studies | Study Design | Risk of bias | Inconsistency | Indirectness | Imprecision | Other consideration | Number of patients | | RR/MD (95% CI) | Certainty | Importance |
| --- | --- | --- | --- | --- | --- | --- | --- | --- | --- | --- | --- | --- | --- |
|  |  |  |  |  |  |  |  |  | Intervention | Comparison |  |  |  |
| NRS | QI 2022 Saxena 2024 | 2 | Randomized trial | Serious^a^ | Serious^b^ | Not serious | Not serious^c^ | None | 66 | 66 | MD=-1.82, 95%CI=-2.1, -1.54 | Low | Crucial |
| VAS | QI 2022 | 1 | Randomized trial | Serious^d^ | Not serious | Not serious | Serious^e^ | None | 51 | 51 | MD=-1.42, 95%CI=-1.83, -1.01 | Low | Important |
| BPI-MSF | Saxena 2024 | 1 | Randomized trial | Serious^f^ | Not serious | Not serious | Very serious^f^ | None | 15 | 15 | MD=-1.46, 95%CI=-2.35, -0.57 | Very low | Important |
| PDQ | Saxena 2024 | 1 | Randomized trial | Serious^f^ | Not serious | Not serious | Very serious^f^ | None | 15 | 15 | MD=-3.00, 95%CI=-5.55, -0.45 | Very low | Important |
| ≥50% responders | Tesfaye 2013 | 1 | Randomized trial | Not serious | Not serious | Serious^g^ | Not serious | None | 86/165 (52.1%) | 64/163 (39.3%) | RR=1.33, 95%CI=1.04, 1.69 | Moderate | Crucial |
| ≥30% responders | Tesfaye 2013 | 1 | Randomized trial | Not serious | Not serious | Serious^g^ | Serious^h^ | None | 102/165 (61.8%) | 91/163 (55.8%) | RR=1.11, 95%CI=0.92, 1.33 | Low | Crucial |
| Any adverse events | QI 2022 Tesfaye 2013 | 2 | Randomized trial | Serious^a^ | Not serious | Not serious | Serious^i^ | None | 70/220 (31.8%) | 64/221 (29.0%) | RR=1.10, 95%CI=0.84, 1.46 | Low | Crucial |
| Somnolence | Saxena 2024 QI 2022 Tesfaye 2013 | 3 | Randomized trial | Serious^a^ | Not serious | Not serious | Very serious^j^ | None | 6/235 (2.6%) | 8/236 (3.4%) | RR=0.79, 95%CI=0.30, 2.08 | Very low | Important |
| Nausea/Vomiting | Saxena 2024 QI 2022 Tesfaye 2013 | 3 | Randomized trial | Serious^a^ | Not serious | Not serious | Very serious^j^ | None | 12/235 (5.1%) | 6/236 (2.5%) | RR=2.02, 95%CI=0.77, 5.27 | Very low | Important |

a. Some studies lack adequate blinding/allocation concealment.

b. Only two trials with differing designs/doses, one trial at high risk of bias, inconsistency cannot be robustly assessed and statistical heterogeneity cannot be reliably estimated with only two studies.

c. Imprecision was not downgraded for NRS because the pooled estimate had a relatively narrow 95% CI and did not cross the line of no effect.

d. Inadequate blinding and/or unclear allocation concealment.

e. Single small study; optimal information size not met and CI is consistent with a range of effects.

f. Extremely small sample size, wide confidence interval, blinding/masking is inadequate, outcome measures may be compromised.

g. The comparison group is high-dose monotherapy, which may limit extrapolation to routine clinical practice.

h. The confidence interval crosses 1 and includes the null effect.

i. Limited studies (2 studies), with confidence intervals covering “possible slight increase or no difference”, making it difficult to rule out clinically important differences.

j. Sparse events and wide confidence intervals.
